# Structure of Exogenous Gene Integration and Event-Specific Detection in the Glyphosate-Tolerant Transgenic Cotton Line BG2-7

**DOI:** 10.1371/journal.pone.0158384

**Published:** 2016-07-05

**Authors:** Xiaobing Zhang, Qiaoling Tang, Xujing Wang, Zhixing Wang

**Affiliations:** 1 Biotechnology Research Institute, Chinese Academy of Agricultural Sciences, Beijing, China; 2 Biology Institute, Hebei Academy of Sciences, Shijiazhuan, China; Nanjing Agricultural University, CHINA

## Abstract

In this study, the flanking sequence of an inserted fragment conferring glyphosate tolerance on transgenic cotton line BG2-7 was analyzed by thermal asymmetric interlaced polymerase chain reaction (TAIL-PCR) and standard PCR. The results showed apparent insertion of the exogenous gene into chromosome D10 of the *Gossypium hirsutum* L. genome, as the left and right borders of the inserted fragment are nucleotides 61,962,952 and 61,962,921 of chromosome D10, respectively. In addition, a 31-bp cotton microsatellite sequence was noted between the genome sequence and the 5' end of the exogenous gene. In total, 84 and 298 bp were deleted from the left and right borders of the exogenous gene, respectively, with 30 bp deleted from the cotton chromosome at the insertion site. According to the flanking sequence obtained, several pairs of event-specific detection primers were designed to amplify sequence between the 5' end of the exogenous gene and the cotton genome junction region as well as between the 3' end and the cotton genome junction region. Based on screening tests, the 5'-end primers GTCATAACGTGACTCCCTTAATTCTCC/CCTATTACACGGCTATGC and 3'-end primers TCCTTTCGCTTTCTTCCCTT/ACACTTACATGGCGTCTTCT were used to detect the respective BG2-7 event-specific primers. The limit of detection of the former primers reached 44 copies, and that of the latter primers reached 88 copies. The results of this study provide useful data for assessment of BG2-7 safety and for accelerating its industrialization.

## Introduction

In agricultural production, genetically modified technology can save in labor costs, improve crop quality, increase production, and reduce pesticide applications. Genetic modifications in agriculture have made important contributions to mitigating the global energy crisis and have helped to address the growing demand for food [1]. However, despite the lack of evidence that genetically modified organisms (GMOs) cause harm to human health or the environment, a variety of controversies have arisen regarding the safety of GMOs [2–4], including biosafety, environmental, and ethical concerns [5–9]. Nonetheless, the global area cultivated with transgenic crops has increased from 1.7 million hectares in 1996 to 200 million hectares in 2015 [10].

Cotton (*Gossypium hirsutum*), an important economic crop for the textile industry, is also grown for food and, in particular, as a source of animal feed in many areas of the world [11]. Currently, cotton is commercially cultivated in more than 70 countries, including the USA, China, and many developing countries. One of the most important goals is to improve the agronomic performance of cotton, especially with respect to resistance to insects and tolerance to herbicides, using transgenic technology. Notably, transgenic herbicide-tolerant cotton provides an excellent approach to weed control for cotton farmers, providing considerable economic and societal benefits [12].

Through transformation of *CP4-epsps*, the Monsanto Company has developed and commercialized a number of transgenic glyphosate-tolerant crop varieties, including genetically modified cotton. Due to patent laws, Monsanto holds a monopoly in relation to global competition, and because Chinese research began after that of Monsanto, no marketable proprietary transgenic glyphosate-resistant cotton varieties have been developed to date in China.

To ensure biosafety and to protect the interests of the import and export trade of agricultural products, countries have established a system of safety assessment, supervision and management of genetically modified crops. Thus, GMO detection technologies have become an important research topic [13–16]. The detection of exogenous components in transgenic crops and their products is mainly concentrated in two areas: exogenous DNA molecules and novel expression of exogenous proteins; typical detection methods for these two types of targets include polymerase chain reaction (PCR) and immunoassay, respectively. PCR is a very effective and reliable method for nucleic acid detection and has the advantages of being rapid and convenient, with high specificity and high sensitivity [17]. Indeed, PCR is the most established and widely used method for detecting genetically modified products [18, 19]. According to the targets to be detected, PCR assays are mainly divided into four types: 1) screening tests, the targets of which are the specific promoter and terminator; 2) gene-specific detection, the targets of which are specific exogenous genes; 3) structure-specific detection, the targets of which are the specific structures of transgenic elements; and 4) event-specific detection, the targets of which are a flanking junction sequence between the exogenous DNA and the plant genome. The flanking junction sequence is particularly important for the identification of different transgenic crop lines. Therefore, event-specific detection is able to quickly and accurately identify different lines of transgenic crops [20].

Many studies have investigated the flanking sequences of insect-resistant transgenic crops [16, 21, 22]. However, there are no reports to date on the flanking sequences of glyphosate-tolerant transgenic cotton from China.

The *G2-aroA* gene (GenBank accession No: EF155478) was identified from the G2 strain of *Pseudomonas fluorescens* isolated from a glyphosate-polluted area. This gene encodes the 445 aa EPSPS protein, which confers glyphosate resistance [23]. The transgenic cotton line BG2-7 containing the glyphosate-tolerance gene *G2-aroA* was generated in our laboratory via *Agrobacterium*-mediated transformation using the upland cotton cultivar Coker 312 as the receptor. Southern blotting showed a single-copy insertion of *G2-aroA* in BG2-7, and an experiment testing glyphosate tolerance indicated that BG2-7 can survive 8000 ppm of the herbicide Roundup (isopropylamine salt of glyphosate as the active ingredient, 41.0%(w/v)) [24]. In the present study, we analyzed the molecular characteristics of BG2-7 and established an event-specific PCR detection system for this line. The results of this study provide useful data for BG2-7 safety assessment and acceleration of the industrialization process of this line.

## Materials and Methods

### Material

*Gossypium hirsutum* L. cv. Coker 312 and the glyphosate-tolerant transgenic cotton line BG2-7 were stored in our laboratory. A Genome Walking Kit, the cloning vector pMD18-T, *rTaq*, *ExTaq* and dNTPs were purchased from TaKaRa Biotechnology (Dalian) Co., Ltd. A DNA marker was purchased from Beijing TransGen Biotech Co., Ltd. Plant DNA Extraction Kit was purchased from TIANGEN Biotechnology (Beijing) Co., Ltd. An Agarose gel purification kit was purchased from Beijing Biomed Gene Technology Co., Ltd. *Escherichia coli* strain DH5α was preserved in our laboratory. All other chemicals were of analytical grade.

### Genomic DNA extraction

Cotton genomic DNA was isolated from the tender leaves of un-transformed control and transgenic cotton plants according to the DNA extraction kit manual. The DNA quality and quantity were based on 260/280-nm and 260/230-nm UV absorption ratios using a spectrophotometer and also analyzed by 1% agarose gel electrophoresis.

### Analysis of the structure of exogenous gene integration in transgenic cotton BG2-7

All primers were designed using Primer3 (http://primer3.ut.ee/) and synthesized by Shanghai Sangon Biotech (China). The primers used in this study are shown in Table 1. The primers for thermal asymmetric interlaced polymerase chain reaction (TAIL-PCR) were designed according to the T-DNA region of the plant expression vector p-*G2-aroA*. The primers Kana-F1, Kana-F2 and Kana-F3 were combined with the left boundary of the T-DNA. TAIL-PCR was conducted according to the protocol of the Genome Walking Kit from TaKaRa. PCR products were sequenced at China Agricultural Crops Research Institute Open Laboratory. The sequences obtained were analyzed against the NCBI database (http://www.ncbi.nlm.nih.gov), the Phytozome database (http://www.phytozome.net/) and the *Gossypium hirsutum* L. database (http://mascotton.njau.edu.cn/html/Data/Genomefhsequence/2015/05/05/16ab0945-19e9-49f7-a09e-8e956ec866bf.html).

**Table 1:** Sequences of primers used in this study.

Primer	Sequence (5ˊ-3ˊ)	Target region	Amplicon (bp)	Reference
*Sad l*-F	CCAAAGGAGGTGCCTGTTCA	Cotton endogene	107	[25]
*Sad I*-R	TTGAGGTGAGTCAGAATGTTGTTC			
G2-F-1	GGCTCCAAATCCATTACCAACC	*G*2-*aroA*	896	This work
G2-R-2	CGCAGGTTCGCCAGTTCA			
Kana-F1	AACACGGCGGCATCAGAGCA	Special primers for Tail-PCR		
Kana-F2	TACCGAGGGGAATTTATGGAACG			
Kana-F3	GTTGCGGTTCTGTCAGTTCCAA			
F1	CTGGCGTAATAGCGAAGAG	Flanking primer of the 3ˊ-terminus	<927	
R1	TTGAAAGACAAGGGATGGA			
F2	CTGGCGTAATAGCGAAGAG		<1770	
R2	AAAGAACTAAACTGGAAACCC			
F3	CCAGCGAGACGAGCAAGA		<848	
R3	ACAAGCGGAGGCGGTATT			
F4	GCAGGAACGCAAACATTG		<1942	
R4	AAAGAACTAAACTGGAAACCC			
F5	AGCCCGATGGCTACTAAG		<1945	
R5	TTGAAAGACAAGGGATGGA			
5ˊ-F1	TGTGGGCCATCGCCCTGATA	Upstream screening primers of the 5ˊ-terminus		
5ˊ-F2	GTTGCGGTTCTGTCAGTTCCAA			
5ˊ-F3	GTCATAACGTGACTCCCTTAATTCTCC			
5ˊ-R1	CCTATTACACGGCTATGC	Downstream screening primers of the 5ˊ-terminus		
5ˊ-R2	CTCAATCAGCCCCAAAAT			
5ˊ-R3	CCACCCATTCCCCAAAGT			
3ˊ-F1	GCTCCTTTCGCTTTCTTCCC	Upstream screening primers of the 3ˊ-terminus		
3ˊ-F2	TCGCTTTCTTCCCTTCCTTT			
3ˊ-F4	AGGGTTCCGATTTAGTGCTT			
3ˊ-F6	TCCTTTCGCTTTCTTCCCTT			
3ˊ-F10	CTAAAAGGCAGGAACGCAAA			
3ˊ-R1	GGTGCATTAAAAGGGTGGCA	Downstream screening primers of the 3ˊ-terminus		
3ˊ-R2	GTGCATTAAAAGGGTGGCAT			
3ˊ-R3	TCTGACATCATGGATCGCAA			
3ˊ-R4	GGCCTCAATTCTCTCCATCA			
3ˊ-R6	CATCAGCACTTTCGAATGCA			
3ˊ-R7	CGTTAACTTCACCGCTCATC			
3ˊ-R8	ACAAGGGATGGACTGTCTTC			
3ˊ-R9	ACATGGCGTCTTCTTCATCT			
3ˊ-R12	ACACTTACATGGCGTCTTCT			

According to the flanking sequence of the left boundary of the T-DNA, upstream primers F1, F2, F3, F4 and F5 were designed for the right boundary of the T-DNA, and downstream primers R1, R2, R3, R4 and R5 were designed for the cotton genome. Standard PCR [21, 26] was conducted using transgenic cotton BG2-7 genomic DNA as the template. The predicted electrophoretic bands were recovered and the gene sequenced, and the flanking sequence of the right boundary of the T-DNA was verified.

### Establishment of the event-specific PCR detection method

#### Primer screening

Upstream and downstream screening primers were designed according to the flanking sequences of the left and right boundaries of the T-DNA; these primers were matched in pairs. Genomic DNA from the wild-type line and transgenic line BG2-7 was used as templates. PCR was conducted with an initial step of 95°C for 5 min, followed by 34 cycles of 95°C for 30 s, 50–65°C for 45 s, and 72°C for 1 min, with a final extension at 72°C for 10 min.

#### Specificity verification

The specificity of selected primers was verified by PCR, which was conducted using genomic DNA isolated from cotton (island cotton 7412, upland cotton K312 and transgenic cotton BG2-7), rice (Zhonghua 11 and TP 309), maize (Zhengdan 958 and Heinuo maize), wheat (Beijing 14 and SALGEM), soybean (R2 and Lingbei 8), and tobacco (NC 89) as templates. The PCR amplification products were detected by 1% agarose gel electrophoresis. The endogenous *Sad l* gene was used to detect the genomic DNA quantity of different cotton varieties.

#### Sensitivity testing

According to mass percentage concentration, 10.00%, 1.00%, 0.50%, 0.10%, 0.05% and 0% solutions of transgenic cotton genomic DNA were prepared with 100 ng/μL genomic DNA from wild-type K312 and 100 ng/μL genomic DNA from transgenic cotton BG2-7. All of the genomic DNA solutions were used as templates. The sensitivity of selected primers was assessed using PCR. The detection method [13] for PCR amplification products was the same as above.

## Results and Discussion

### Determination and analysis of the flanking sequences of transgenic cotton BG2-7

#### Determination of the flanking sequence of the 5ʹ-terminus of the exogenous gene

The flanking sequences of the 5ʹ-terminus of the inserted gene were obtained by TAIL-PCR amplification and verified by PCR. The flanking sequences were aligned with the known vector sequence, showing overlapping sequences of 431 bp with a similarity of 100%. The remaining sequence is an unknown sequence of approximately 734 bp. According to a blastn search, 626 bp of the sequence exhibits 94.2% similarity to the sequence of *Gossypium raimondii* chromosome 11, with 97.0% similarity to *Gossypium hirsutum* L. chromosome D10.

#### Determination of the flanking sequence of the 3ʹ-terminus of the exogenous gene

We also attempted to amplify the flanking sequence of the 3ʹ-terminal end of the inserted gene using TAIL-PCR and primers including the 3ʹ-terminal special primer designed according to the right boundary of the T-DNA and arbitrary primers from the genome walking kit. However, specific bands were not identified. The reason for this may be related to the specificity of the designed primers and the particular complexity of the allotetraploid genome of upland cotton (*Gossypium hirsutum* L.) [27].

The following method was adopted to analyze the flanking sequence of the 3ʹ-terminus of the inserted DNA. Based on the known sequence of the 5ʹ-terminus of the inserted sequence, the partial DNA sequence of the *Gossypium raimondii* genome from 61,252,000 to 61,253,972 of chromosome 11 were downloaded from Phytozome. Upstream primers were designed for the 3ʹ-terminal boundary of the exogenous DNA, and downstream primers were designed for the downloaded cotton genome sequence. Standard PCR was then performed using BG2-7 genomic DNA as the template. According to gel electrophoresis, there were anticipated objective strips in size among the obtained electrophoresis strips. The corresponding bands were recovered, ligated to the T-vector, transformed into *E*. *coli*, sequenced, and aligned. Ultimately, the 3ʹ-terminal flanking sequence was identified.

#### Analysis of insertion structure characteristics

**Fig 1:**
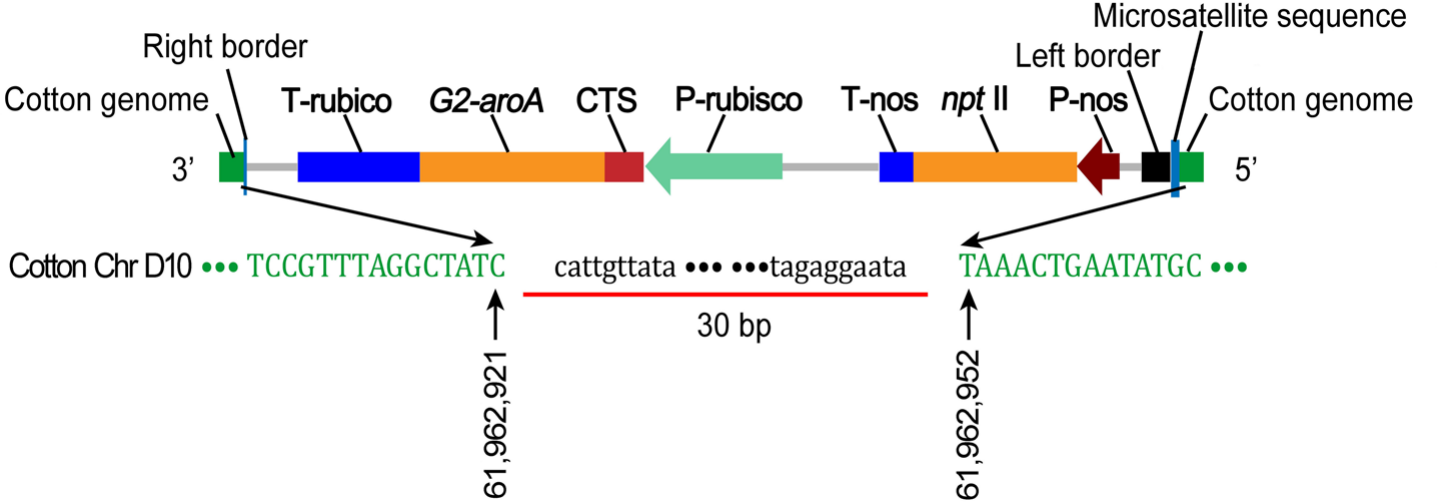
A putative integration structure diagram of the exogenous gene insertion. P-rubisco: Daisy Rubisco small subunit promoter; T-rubisco: Daisy Rubisco small subunit terminator; CTS: Daisy Rubisco small subunit chloroplast signal peptide; P-nos: Nos promoter; T-nos: Nos terminator; *npt* II: neomycin phosphotransferase gene; *G2*-*aroA*: 5-enolpyruvyl-shikimate-3-phosphate synthase gene. Note: The underline represents the missing bases.

The cotton genome sequence on both sides of the exogenous inserted DNA were obtained via amplification of the 5ʹ-terminal flanking sequence of the inserted gene according to the genome walking method and amplification of the 3ʹ-terminal flanking sequence using standard PCR. The results of a bioinformatic-based comparison indicate that the exogenous DNA is inserted into chromosome D10 (Fig 1). Specifically, the 5ʹ-terminus of the T-DNA is inserted at position 61,962,952 of cotton chromosome D10. Further analysis reveals that 81 bp are deleted from the left boundary of the 5ʹ-terminal end of the exogenous sequence; 31 bp (CTGCTTTTCAAGAGGTCGGTGCATTAAAAGG) form a microsatellite DNA sequence between the left boundary of the 5ʹ-terminal end of the exogenous sequence and the cotton genome. The 3ʹ-terminal end of the exogenous DNA is inserted at position 61,962,921 of cotton chromosome D10, with deletion of 298 bp from the right boundary of the T-DNA and 30 bp of cotton chromosome D10.

It is generally accepted that the process of *Agrobacterium* T-DNA integration into the cotton genome is similar to gene recombination [28]. Part of the sequence of the T-DNA left border was deleted via the integration of the exogenous gene. In addition, a microsatellite DNA sequence (filler DNA), which was produced due to the low accuracy of DNA repair, was inserted between the 5ʹ-terminal sequence of the exogenous gene and the cotton chromosome, similar to the reported structure of the left border junction region of an *Arabidopsis thaliana* transformant [28,29]. The sequence of the T-DNA right border was completely deleted, similar to the structure of the right border junction region of co-transformed rice [30]. The reason for the above results may be associated with DNA repair factors or replication enzymes in cotton that are not exactly identical to those in rice or *Arabidopsis thaliana*.

### Establishment of an event-specific PCR detection method

**Fig 2:**
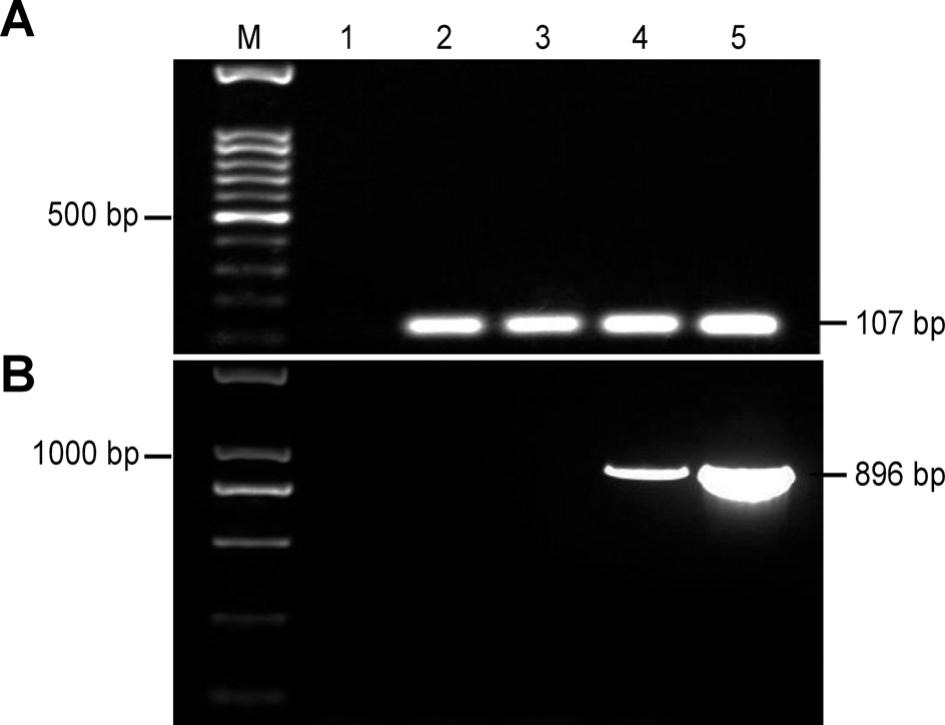
PCR amplification of the positive control. Note: (a) Amplification results for the cotton endogenous reference gene *Sad 1*; M: 100-bp marker. (b) Amplification results for the cotton exogenous gene *G2*-*aroA*; M: Trans2K^TM^ DNA marker. 1: ddH_2_O; 2: Island cotton 7124; 3: Upland cotton K312; 4: Transgenic cotton BG2-7; 5: Plasmid DNA.

To ensure the repeatability and credibility of developed PCR systems, positive controls for the endogenous reference gene and exogenous gene are necessary [13]. The *Sad l* gene is specific in different species but exhibits low heterogeneity among cotton cultivars. Two copies of the *Sad l* gene are present in the haploid cotton genome [25]. A primer pair of *Sad l*-F/*Sad l*-R for the endogenous *Sad l* gene was selected and successfully used to amplify a 107-bp fragment using DNA from island cotton 7124, upland cotton K312, and transgenic cotton BG2-7 as well as plasmid DNA. However, no fragment was amplified from the negative control, i.e., double-distilled water (Fig 2(A)), suggesting that the various genomic DNA extracted was completely suitable for PCR. The primer pair of G2-F-1/G2-R-2 for the exogenous gene *G2-aroA* was used to amplify an 896-bp fragment from the T-DNA of transgenic cotton BG2-7 and plasmid DNA, whereas no fragment was amplified from the other samples (Fig 2(B)). According to the tests for the cotton endogenous reference gene *Sad l* and the exogenous gene *G2-aroA*, factors, including DNA quality and experimental operation, that might affect the reliability of the results or false-negative results were excluded.

To establish an event-specific PCR detection system, primer pairs should be designed based upon the specific sequences of the integrated DNA fragment of the flanking region. For each pair of primers, one primer was located in the cotton genome and the other on the T-DNA. Both upstream and downstream primers were designed, and through one-to-one combinatorial screening, primer pair 5ʹ-F3/5ʹ-R1 was selected as the 5ʹ-terminal flanking sequence-specific detection primers and primer pair 3ʹ-F6/3ʹ-R12 as the 3ʹ-terminal flanking sequence-specific detection primer. These primer pairs were chosen because their amplification efficiency and specificity were better than other combinations. The size of the 5ʹ-terminus amplified target fragment was 645 bp, and the size of other product was 642 bp. Simplex PCR for the 5ʹ-terminus and other terminus-specific detection was conducted at annealing temperatures of 55°C and 58°C, respectively.

**Fig 3:**
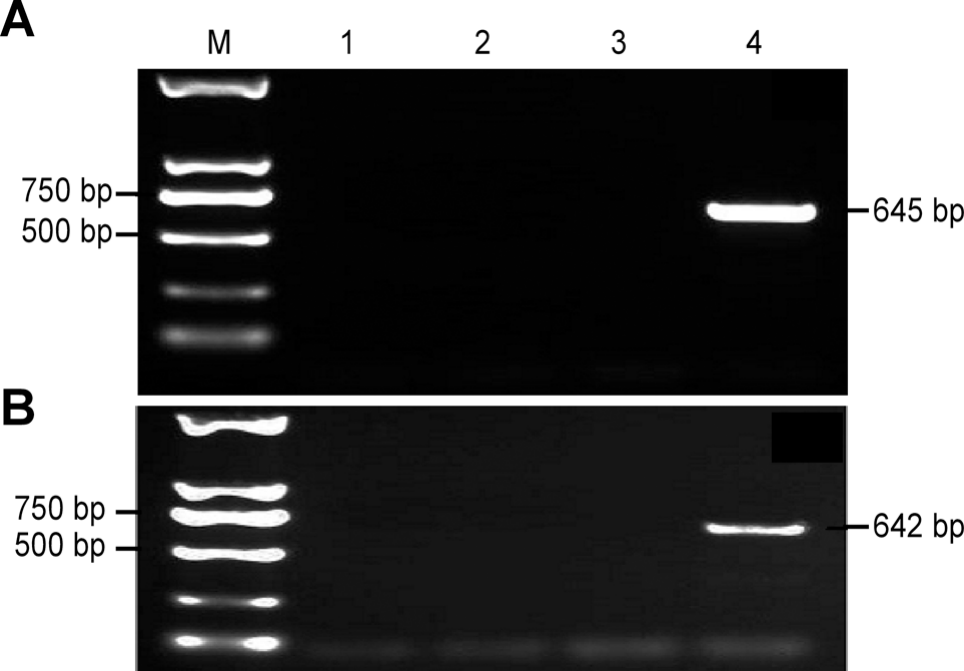
**Amplification results for intraspecific specific qualitative PCR using 5ʹ-F3/5ʹ-R1 (a) and 3ʹ-F6/3ʹ-R12 (b).** M: Trans2K^TM^ DNA marker; 1: ddH_2_O; 2: Island cotton 7124; 3: Upland cotton K312; 4: Transgenic cotton BG2-7.

**Fig 4:**
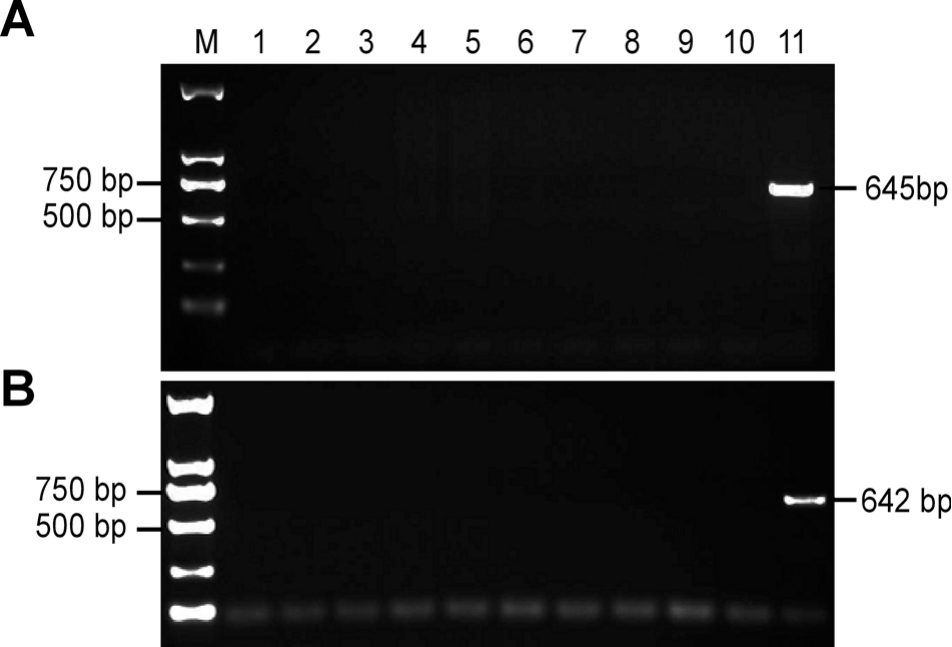
**Amplification results for interspecific specific qualitative PCR using 5ʹ-F3/5ʹ-R1 (a) and 3ʹ-F6/3ʹ-R12 (b).** M: Trans2K^TM^ DNA marker; 1: ddH_2_O; 2: Zhonghua 11 (rice); 3: TP309 (rice); 4: Zhengdan 958 (maize); 5: Heinuo maize (maize); 6: Beijing 14 (wheat); 7: SALGEM (wheat); 8: R2 (soybean); 9: Lingbei 8 (soybean); 10: NC89 (tobacco); 11: Transgenic cotton BG2-7.

The intraspecific specificity of primers 5ʹ-F3/5ʹ-R1 and 3ʹ-F6/3ʹ-R12 was verified using genomic DNA from different cotton varieties, and the expected 645-bp and 642-bp fragments, respectively, were amplified only using DNA from transgenic cotton line BG2-7 (Fig 3). The interspecific specificity of primers 5ʹ-F3/5ʹ-R1 and 3ʹ-F6/3ʹ-R12 was verified using genomic DNA from different crops including rice, maize, wheat, soybean and tobacco; except for transgenic cotton, no band was amplified from these samples (Fig 4). Therefore, these two primer pairs can be successfully used to discriminate a single transgenic cotton variety from other samples. This result was mainly attributed to the specificity of the primer pairs designed to amplify specific fragments of the flanking region of the insertion site.

**Fig 5:**
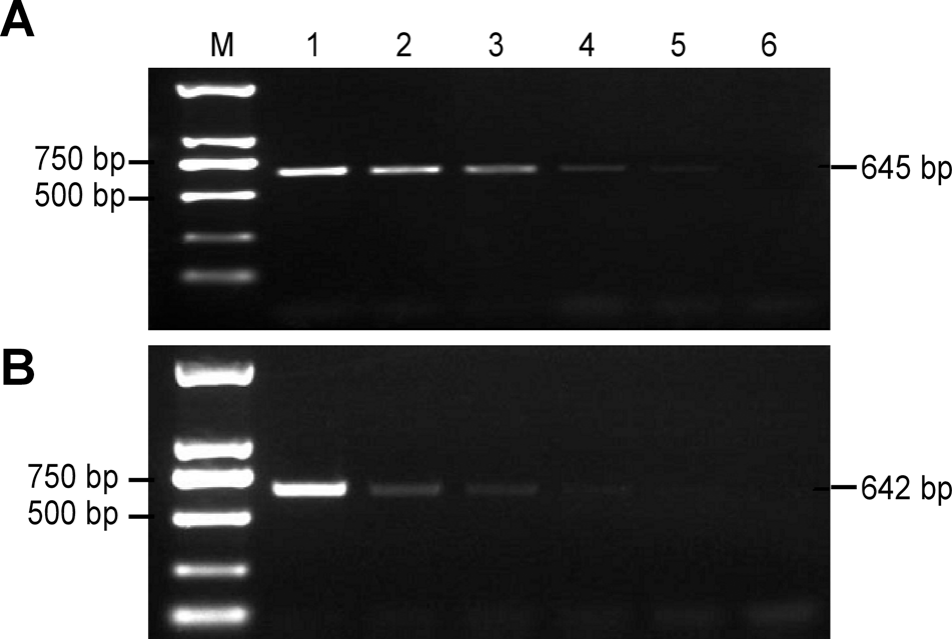
**Sensitivity tests for the event-specific primer pairs 5ʹ-F3/5ʹ-R1 (a) and 3ʹ-F6/3ʹ-R12 (b).** M: Trans2K^TM^ DNA marker; 1–6: The contents of the transgenic cotton BG2-7 genome DNA were 10.00%, 1.00%, 0.50%, 0.10%, 0.05% and 0%, respectively.

Because degraded or a low amount of DNA derived from transgenic feed or food is often the only material available for practical detection applications, a PCR detection system must have high sensitivity [13]. To test the limits of the established qualitative PCR detection systems, mixed DNA solutions were used as templates. In these samples, the concentrations of transgenic cotton BG2-7 genomic DNA were 10%, 1%, 0.50%, 0.10%, 0.05% and 0%. The results indicate that the 645-bp DNA target fragments of the 5ʹ-terminal event-specific primers were detected at all five levels, except for the negative control, with approximately 44 haploid genome copies (Fig 5(A)). In addition, the 642-bp DNA target fragment of the 3ʹ-terminal event-specific primers were detected at the levels tested, with the exception of the 0.05% level and the negative control, with approximately 88 haploid genome copies (Fig 5(B)). The above findings indicate that the established qualitative PCR detection systems are suitable for the practical detection of transgenic cotton BG2-7 samples. The detection limits were acceptable and met the labeling requirements of regulations in China, the EU (0.9%), Korea (5.0%), and Japan (3.0%) [13].

Quantitative detection of genetically modified samples is also very important, and event-specific PCR primer pairs that are not only suitable for qualitative detection but can also be applied for quantitative determination are optimal for genetically modified samples. Unfortunately, the most ideal primers were not obtained by screening. Fortunately, the primer pair that was used to determine the copy number of the *G2-aroA* gene in transgenic cotton using quantitative real-time PCR (data not shown) can also be applied for the quantitative detection of transgenic cotton BG2-7.

## Conclusions

In conclusion, the flanking sequence of an inserted exogenous fragment in BG2-7 was analyzed by TAIL-PCR and standard PCR. According to the flanking sequence obtained, event-specific conventional PCR systems were established that could be successfully used for detecting transgenic cotton BG2-7, providing useful data for BG2-7 safety assessment.

## Supporting Information

S1 Fig The 5´-terminus flanking sequences from transgenic cotton BG2-7. (TIF)

S2 Fig The 3´-terminus flanking sequences from transgenic cotton BG2-7. (TIF)

S3 Fig The sequences of verification test from K312. (TIF)

S1 File The flanking sequences of the exogenous gene. (DOC)
