# Supplementary figures and images for: Structure of Exogenous Gene Integration and Event-Specific Detection in the Glyphosate-Tolerant Transgenic Cotton Line BG2-7
Source: PLoS One. 2016 Jul 5;11(7):e0158384. doi: 10.1371/journal.pone.0158384 (PMC4933378; doi:10.1371/journal.pone.0158384)

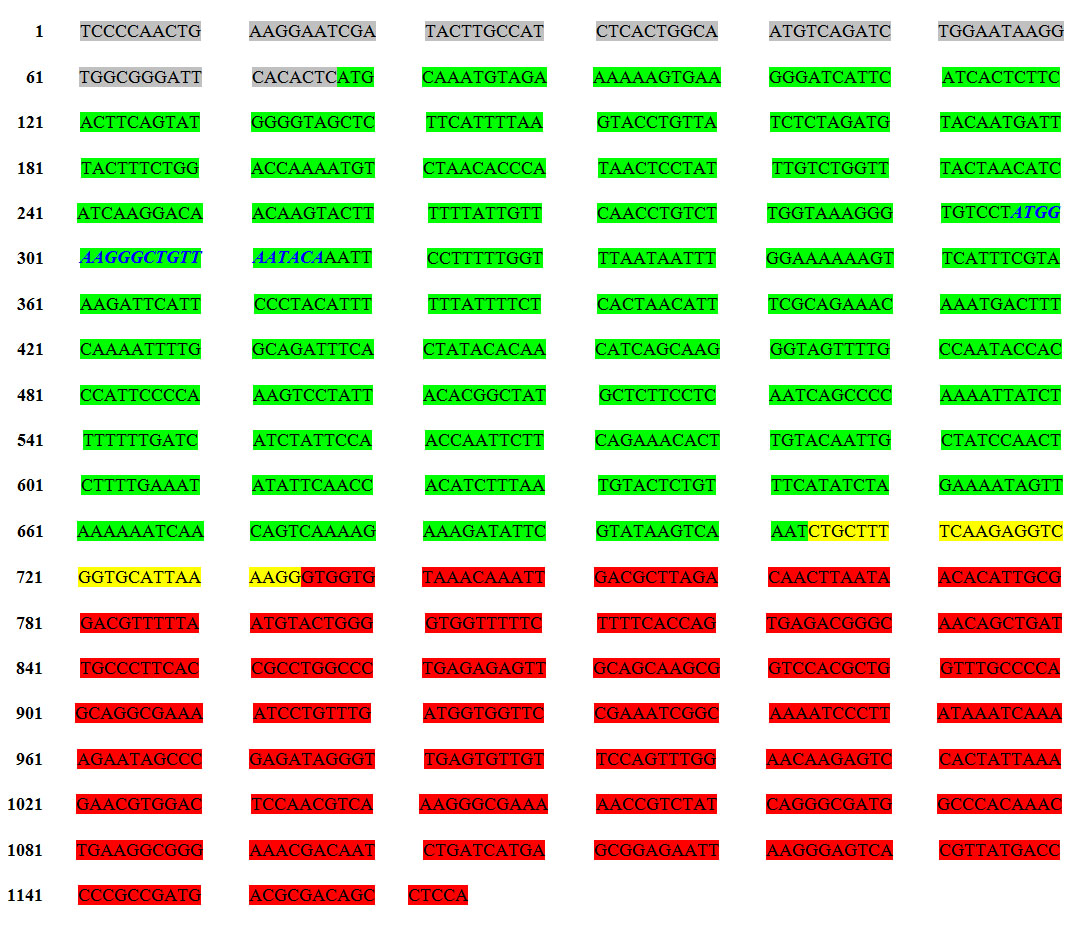

Supplement: S1 Fig — (TIF) [file pone.0158384.s001.tif]

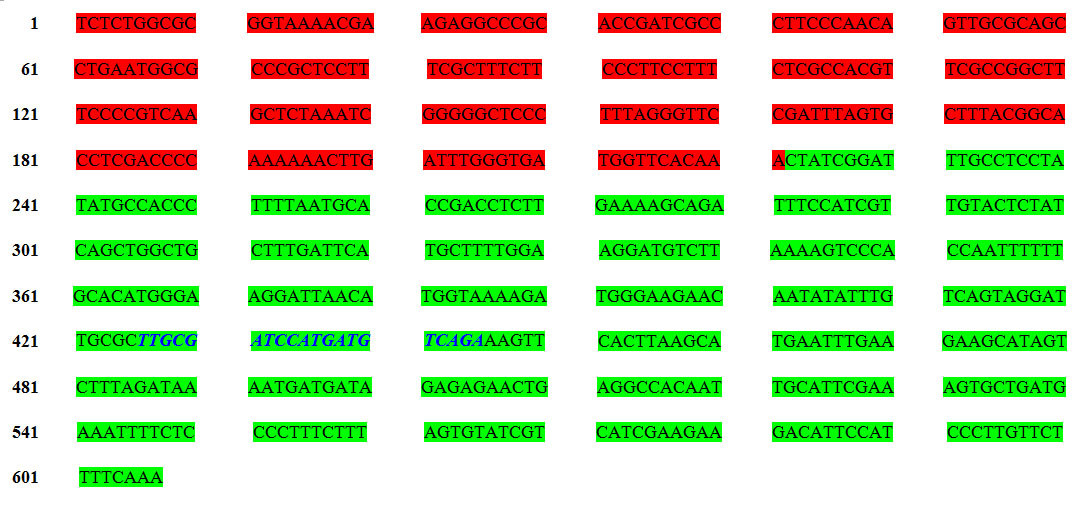

Supplement: S2 Fig — (TIF) [file pone.0158384.s002.tif]

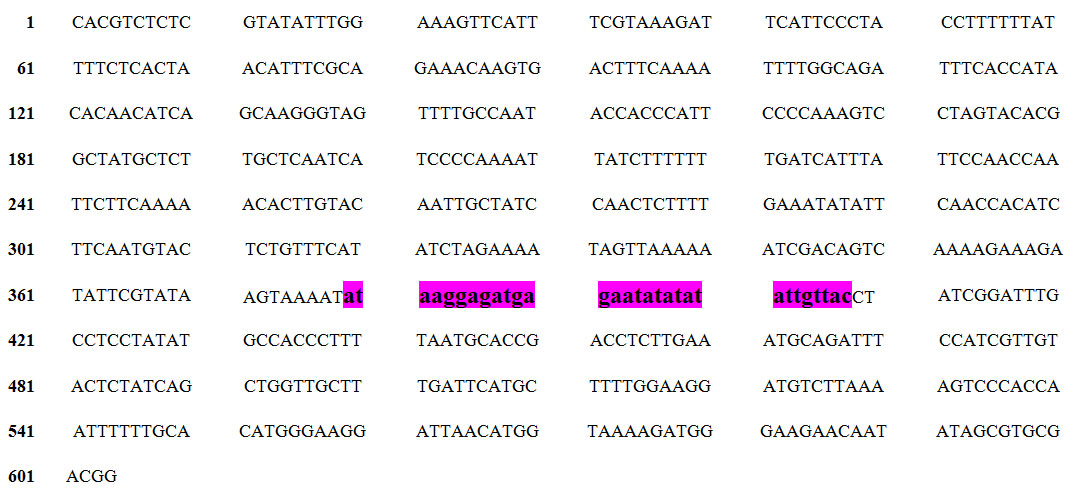

Supplement: S3 Fig — (TIF) [file pone.0158384.s003.tif]
