# Supplementary material for: Structure of Exogenous Gene Integration and Event-Specific Detection in the Glyphosate-Tolerant Transgenic Cotton Line BG2-7
Source: PLoS One. 2016 Jul 5;11(7):e0158384. doi: 10.1371/journal.pone.0158384 (PMC4933378; doi:10.1371/journal.pone.0158384)
Supplement: S1 File — (DOC) [file pone.0158384.s004.doc]

**Supporting Information**

**The flanking sequences of the 5´-terminus of the exogenous gene**

The flanking sequences of the 5´-terminus of the inserted gene obtained by TAIL-PCR were shown in S1 Fig.

**S1 Fig. The 5´-terminus flanking sequences from transgenic cotton BG2-7**

Note: The gray background represents the sequences of the sequencing vector, and the green background represents cotton genome sequences, and the yellow background represents a microsatellite DNA sequences, and the red background represents expression vector sequences. These blue italic characters represent a primer named F that is used in a validation test.

**The flanking sequences of the 3´-terminus of the exogenous gene**

The flanking sequences of the 3´-terminus of the inserted gene obtained by standard PCR were shown in S2 Fig.

**S2 Fig. The 3´-terminus flanking sequences from transgenic cotton BG2-7**

Note: The red background represents expression vector sequences, and the green background represents cotton genome sequences. These blue italic characters represent a primer named R that is used in a validation test.

**Verification of the flanking sequences**

Based on the known flanking sequences of the 5´- and 3´-terminus the inserted sequence, upstream primer named F (ATGGAAGGGCTGTTAATACA) were designed for the 5´-terminus flanking sequence, and downstream primers named R (TCTGACATCATGGATCGCAA) were designed for the 5´-terminus flanking sequence. Standard PCR was performed using K312 genomic DNA as the template. The electrophoretic band was recovered and sequenced. According to a blastn search, 604 bp of the sequence obtained (S3 Fig) exhibits 98% similarity to the sequence of *Gossypium hirsutum* L. chromosome D10.

**S3 Fig. The sequences of verification test from K312**

Note: Pink background lowercase characters represent the putative missing bases
